# NAT10 regulates neutrophil pyroptosis in sepsis via acetylating ULK1 RNA and activating STING pathway

**DOI:** 10.1038/s42003-022-03868-x

**Published:** 2022-09-06

**Authors:** Hao Zhang, Zhaoyuan Chen, Ji’an Zhou, Jiahui Gu, Han Wu, Yi Jiang, Shenjia Gao, Yun Liao, Ruling Shen, Changhong Miao, Wankun Chen

**Affiliations:** 1 Department of Anesthesiology, Zhongshan Hospital, Fudan University; Cancer Center, Zhongshan Hospital, Fudan University, 200032 Shanghai, China; 2 Shanghai Key laboratory of Perioperative Stress and Protection, Shanghai, China; 3 Department of Respiratory and Critical Care Medicine, Huadong Hospital Affiliated to Fudan University, Shanghai, China; 4 Shanghai Medical College of Fudan University, Shanghai, China; 5 Shanghai Laboratory Animal Research Center, 201203 Shanghai, China; 6 Fudan Zhangjiang Institute, Shanghai, 201203 China

**Keywords:** Bacterial infection, Cell death and immune response

## Abstract

Emerging evidence suggests that pyroptosis is involved in sepsis. However, the role of neutrophil pyroptosis in sepsis and the mechanisms remains elusive. We find that N-acetyltransferase 10 (NAT10), an acetyltransferase responsible for the N^4^-acetylation of Cytidine (ac^4^C) in mRNA, is significantly downregulated in neutrophils from septic mice. Neutrophil-specific over-expression of NAT10 improves the survival and ameliorates lung injury in septic mice by inhibiting neutrophil pyroptosis. Notably, UNC-52-like kinase 1 (ULK1) is identified as the target of NAT10 in neutrophils. The decreased expression of NAT10 resultes in the decay of ULK1 transcripts and therefore the reduced expression of ULK1. As a regulator of STING phosphorylation, the loss of ULK1 enhances the activation of STING-IRF3 signaling and subsequently the elevated pyroptosis-inducing NLRP3 inflammasome in neutrophils. While over-expression of NAT10 restrains pyroptosis in neutrophils as well as septic lethality in mice by reversing the ULK1-STING-NLRP3 axis. The decreased expression of NAT10 are also observed in sepsis patients and its correlation with clinical severity is found. Collectively, our findings disclose that NAT10 is a negative regulator of neutrophil pyroptosis and its downregulation contributes to the progress of sepsis by exacerbating pyroptosis via the ULK1-STING-NLRP3 axis, therefore revealing a potential therapeutic target for sepsis.

## Abstract (web summary)

The enzyme N-acetyltransferase NAT10 is a negative regulator of neutrophil pyroptosis and its downregulation contributes to the progress of sepsis by exacerbating pyroptosis via the ULK1-STING-NLRP3 pathway.

## Introduction

Sepsis is a life-threatening complication caused by pathogen infection, which remains the leading cause of death in the intensive care units (ICUs)^1^. Emerging evidence have indicated that extensive immune cell deaths is a major driver of immune dysfunction and the development of sepsis. It has been reported that progressive T cell apoptosis was associated with poor outcome in sepsis patients^2^. Ferroptosis has been found in dendritic cells in septic mice and the inhibition of ferroptosis can relieve sepsis^3^. NETosis, a form of neutrophil death, is also involved in the development of multiple organ failure in sepsis^4^. Recently, pyroptosis has just become another type of immune cell deaths of interest in sepsis, despite knocking out of one of the pyroptotic mediators, caspase-1, has long been known to induce resistance to endotoxic shock in mice^5^.

As the first line of host defense against infection and the critical regulator of innate and adaptive immune cells, neutrophils play crucial roles in the pathogenesis of sepsis^6,7^. Despite most of the literature on pyroptosis has focused on monocytes and macrophages, pyroptosis in neutrophils are getting more and more attention. It is now observed that some neutrophils will undergo pyroptosis and mediate the inflammatory response during infection^8^, and assumed as a major pathological factor in sepsis^9^. However, there are still few studies on the upstream molecules of pyroptosis in sepsis at present. Therefore, the field still has gaps in knowledge regarding the regulatory mechanisms of neutrophil pyroptosis and in sepsis.

Epitranscriptomic modifications are important mechanisms involved in gene expression by regulating mRNA processing events, including splicing, transport, translation, and turnover^10^. Among which, N^4^-acetylation of Cytidine (ac^4^C) catalyzed by the *N*-acetyltransferase 10 (NAT10) regulates mRNA stability and translation^11^. NAT10 participates in multiple cellular processes via its acetyltransferase activity, such as cell deaths including apoptosis and autophagy^12,13^. Remarkably, the prognostic and immunological role of NAT10 has been observed in most cancers, such as hepatocellular carcinoma^14^, gastric cancer^15^, and leukemia^16^; either by enhancing cancer cell proliferation, facilitating cancer metastasis or altering metabolism et al. Interestingly, NAT10 genetic depletion or its chemical inhibition by the compound Remodelin has been reported to enhance healthspan in aging mouse model^17^. Moreover, aberrant NAT10 and the ac^4^C levels also contributes to infectious diseases including HIV infection^18^ and chronic respiratory tract infections^19^. All these studies demonstrate the multi-functionality of NAT10. Nevertheless, the role of epitranscriptomic modifications in sepsis pathology and neutrophil pyroptosis is largely unknown.

Recent study suggested that Pathogen (PAMPs) and danger associated molecular patterns (DAMPs) can activate the STING (Stimulator of Interferon Genes) pathway and furthermore, the hyperactivation of STING signaling leads to lethal sepsis by inducing over-exuberant inflammation^20^. Upon stimulation, STING was perinuclearly translocated and further bound to Type-I interferons (IFN) regulatory factor 3 (IRF3) and phosphorylated IRF3 to activate the downstream functional signaling pathways. Intriguingly, UNC-52-like kinase 1 (ULK1), which was first identified as important components of the autophagy process^21^, has recently been found regulated by NAT10, and been reported to suppress the activation of STING pathway by inhibitory phosphorylation^22^. Together these recent data give us hints about the potential regulatory network between NAT10, ULK1, and the STING signaling pathway.

Here, we demonstrated that NAT10 is an important negative regulator of neutrophil pyroptosis, whose expression remarkably decreased in sepsis and contributed to the excessive pyroptosis in sepsis neutrophils. Neutrophils-specific over-expression of NAT10 improved the survival and ameliorated lung injury in sepsis mice by diminishing pyroptosis. Importantly, ULK1 was identified as the target of NAT10 in neutrophils. Over-expression of NAT10 upregulated the expression of ULK1, and hence, the impaired activation of STING signaling and subsequently the restrained pyroptosis-inducing NLRP3 inflammasome in neutrophils. Therefore, the loss of NAT10 exacerbated pyroptosis in neutrophils as well as septic lethality while over-expressing NAT10 improved survival and attenuated lung injury in septic mice. Clinically, the decreased expression of NAT10 and its correlation with clinical severity were also observed in sepsis patients. Thus, our findings uncover a critical mechanism that regulates neutrophil pyroptosis and indicate the potential of neutrophils and NAT10 as cellular and molecular targets in sepsis intervention.

## Results

### Pyroptosis occurs in neutrophils of septic mice

**Fig. 1:**
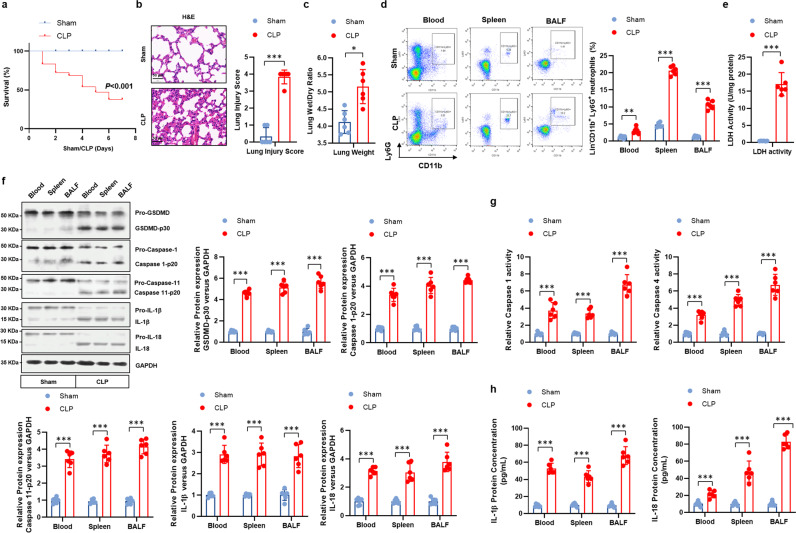
Pyroptosis is induced in neutrophils in septic mice. C57 BL/6 mice were randomly assigned to sham or CLP-induced sepsis group, *n* = 20 per group for the survival analysis, *n* = 6 per group for other experiments. **a** Survival of sham and CLP-induced septic mice. **b** For pulmonary histology analysis, murine lungs were harvested. Lung injury was assessed by H&E staining. Lung injury score was recorded (Scale bar, 50 μm). **c** Wet/dry ratio of lung tissues were calculated. **d** CD11b^+^Ly6G^+^ neutrophils in the peripheral blood, spleens, and BALF of sham and CLP mice were detected by flow cytometry. **e** LDH activity was determined in neutrophils to indicate cell death. **f** Pyroptosis-related proteins were detected in the neutrophils isolated from sham or CLP-induced septic mice by western blot. **g** Activity of caspase 1 and caspase 4 in neutrophils of sham or CLP-induced septic mice were measured by colorimetric assay. **h** Concentration of IL-1β and IL-18 in the supernatant of neutrophil cultures were assessed by ELISA. Experiments were repeated for three times independently and representative image from one of the experiments were shown. Data are presented as dot-plots of individual experiments and mean values ± SD. *P < 0.05, **P < 0.01, ***P < 0.001.

To determine the pyroptosis of neutrophils in sepsis, we utilized a murine model of CLP-induced sepsis, with a survival rate ~40% after 8 days (Fig. 1a) and increased serum levels of inflammatory cytokines (Supplementary Fig. 1a). Compared to the sham group, septic mice exhibited profound lung injury (Fig. 1b), which was further indicated by higher wet to dry weight ratio of lung tissues (Fig. 1c). Injuries were also observed in the kidneys and livers of CLP- induced septic mice (Supplementary Fig. 1b–g). To gain a better understanding of the relationship between pyroptosis and sepsis, pyroptosis inhibitor Ac-FLTD-CMK was administered to sham or CLP-induced septic mice and the disease severity was assessed. As shown by the increased survival rate (Supplementary Fig. 2a), the ameliorated lung injury (Supplementary Fig. 2b, c) and the reduced inflammatory cytokines (Supplementary Fig. 2d) in pyroptosis-deficient CLP mice compared to vehicle mice, it suggests a critical role of pyroptosis in sepsis. Unsurprisingly, increased proportion of CD11b^+^Ly6G^+^ neutrophils were observed in the blood, spleens and BALF of septic mice, compared to the sham group (Fig. 1d). However, elevated cell death of neutrophils were observed in CLP mice as indicated by the LDH assay, compared to the sham mice (Fig. 1e). Pyroptosis is characterized by the expression level of pyroptosis-related proteins, including the cleaved Gasdermin D (GSDMD-p30), caspase 1 and caspase 11, the activity of caspase 1 and caspase 4, and the production of inflammatory cytokines L-1β and IL-18^23^. Thus, as suggested by the data (Fig. 1f–h), neutrophils in septic mice exhibited distinct characteristics of pyroptosis.

### The expression of NAT10 decreases in septic neutrophils

**Fig. 2:**
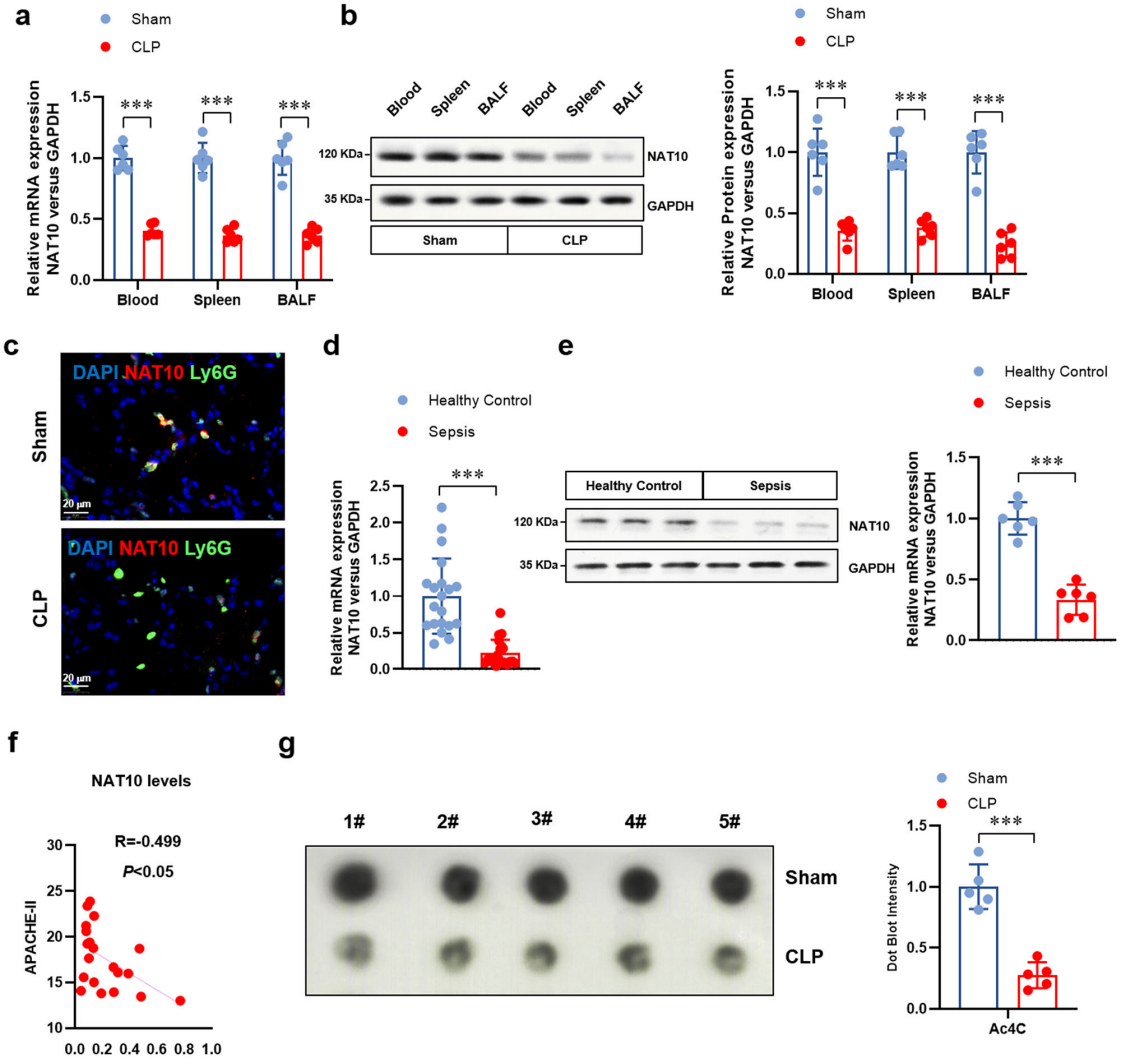
The expression of NAT10 decreased in neutrophils from septic mice and patients. Protein and mRNA were collected from neutrophils derived from peripheral blood, spleens, and BALF of sham or CLP-induced septic mice (*n* = 6 per group) and from BALF neutrophils of healthy control or sepsis patients (*n* = 20 per group). **a** The mRNA expression of *NAT10* in neutrophils from peripheral blood, spleens, and BALF of sham or CLP-induced septic mice were detected by real-time PCR. **b** The protein expression of NAT10 in neutrophils from peripheral blood, spleens, and BALF of sham or CLP-induced septic mice were detected by western blot. **c** The expression of NAT10 in neutrophils (Ly6G^+^) from lung tissues of sham or CLP-induced septic mice was detected by immunofluorescence (Scale bar, 20 μm). Blue: DAPI, red: NAT10, green: Ly6G. **d** The mRNA expression of *NAT10* in neutrophils from BALF of healthy control or sepsis patients were detected by real-time PCR. **e** The protein expression of NAT10 in neutrophils from BALF of healthy control or sepsis patients were detected by western blot. **f** The correlation between mRNA expression level of *NAT10* in BALF neutrophils from sepsis patients and the Acute Physiology and Chronic Health Status Scoring System II (APACHE II) score was determined (*P* = 0.0251). **g** Ac^4^C level in RNA of BALF neutrophils from sham or PICS mice were detected by dot blot. Experiments were repeated for 3 times independently and representative image from one of the experiments were shown. Data are presented as dot-plots of individual experiments and mean values ± SD. **P < 0.01, ***P < 0.001.

To investigate whether NAT10 has been involved in the regulation of neutrophil-pyroptosis and sepsis development, the expression of NAT10 was measured in septic mice and patients in the first place. Unexpectedly, we found a remarkably decreased expression level of NAT10 in neutrophils from blood, spleens, and BALF of septic mice, compared to the sham mice (Fig. 2a, b). Immunofluorescence assay also verified the lower level of NAT10 in neutrophils of septic lungs (Fig. 2c). BALF neutrophils from sepsis patients and healthy controls were then collected and NAT10 expression was detected. Consistently, NAT10 was found significantly expressed lower in neutrophils of sepsis patients, compared to healthy controls (Fig. 2d, e). More importantly, the low expression of NAT10 was found positively correlated with the clinical severity, as indicated by the APACHE II score (Fig. 2f). In some extent, this gives us hints on the participation of NAT10 in sepsis development. Given that NAT10 is an acetyltransferase responsible for ac^4^C of target RNAs, we then assessed NAT10-dependent ac^4^C level. Compared to the sham mice, RNA ac^4^C abundance in BALF neutrophils of septic mice distinctly reduced (Fig. 2g), which is in accordance with the decreased expression of NAT10. Accordingly, these data reveal the decreased expression pattern of NAT10 and its specific RNA modification ac^4^C in sepsis neutrophils.

### Neutrophils specific NAT10 over-expression improves survival and ameliorates lung injury in septic mice by diminishing neutrophil pyroptosis

**Fig. 3:**
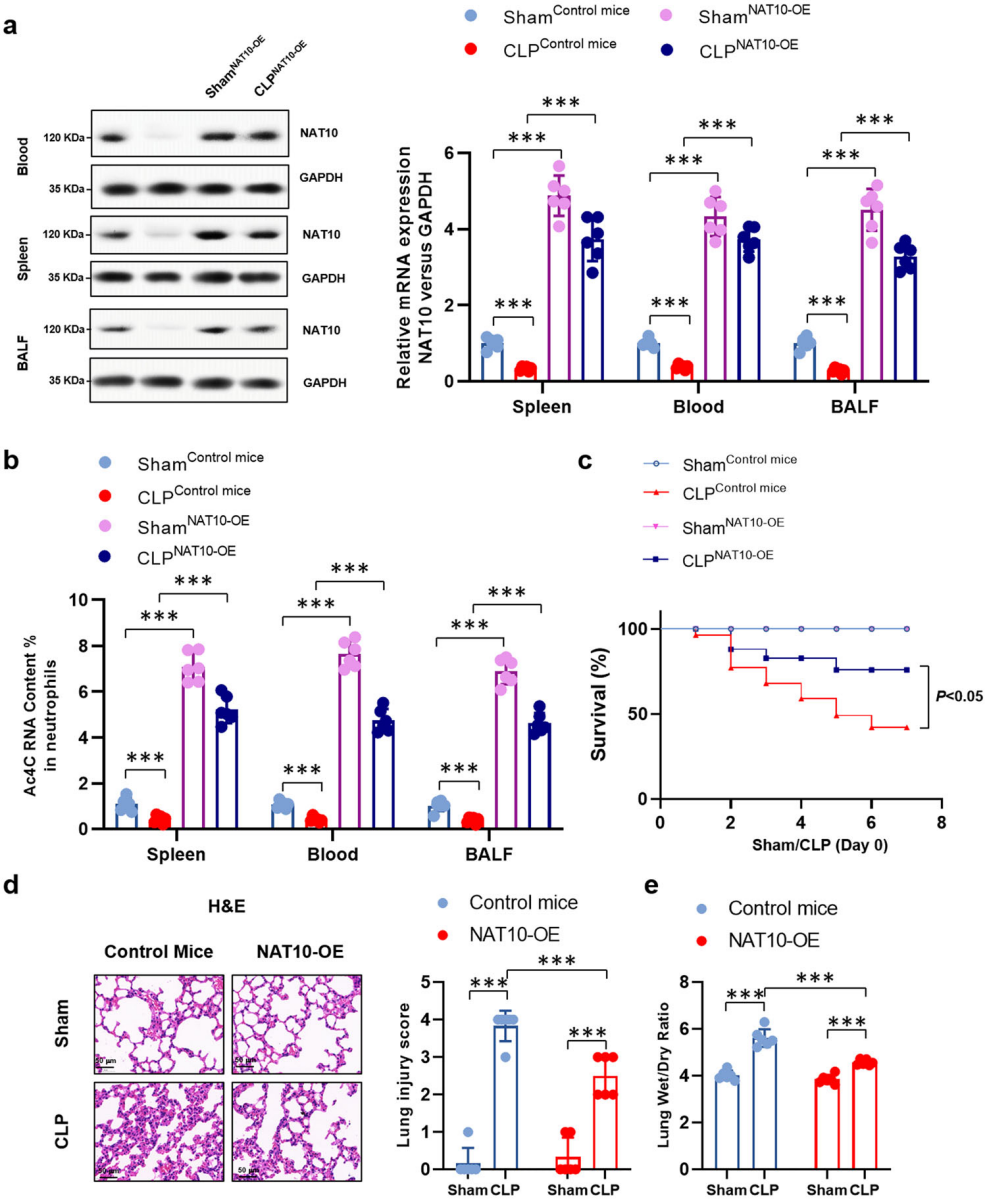
Neutrophils specific NAT10 over-expression improved survival and ameliorated lung injury in septic mice. Transgenic mice with neutrophil-specific over-expression of NAT10 (MRP8Cre/Rosa26-loxP-stop-loxP-NAT10, NAT10-OE) or control mice (Rosa26-loxP-stop-loxP-NAT10, NAT10-Ctrl) were randomly assigned to the sham or CLP-induced sepsis group. *n* = 6 per group. **a** Protein level of NAT10 in peripheral blood, spleens and BALF neutrophils was determined by western blot. **b** RNA ac^4^C abundance in peripheral blood, spleens, and BALF neutrophils was determined by ac^4^C RIP RNA quantification. **c** Survival of mice were monitored. **d** Lungs were harvested and H&E staining was conducted to indicate lung injury. Lung injury score was recorded (Scale bar, 50 μm). **e** Wet/dry ratio of lung tissues were calculated. Experiments were repeated for three times independently and representative image from one of the experiments were shown. Data are presented as dot-plots of individual experiments and mean values ± SD. *P < 0.05, **P < 0.01, ***P < 0.001.

**Fig. 4:**
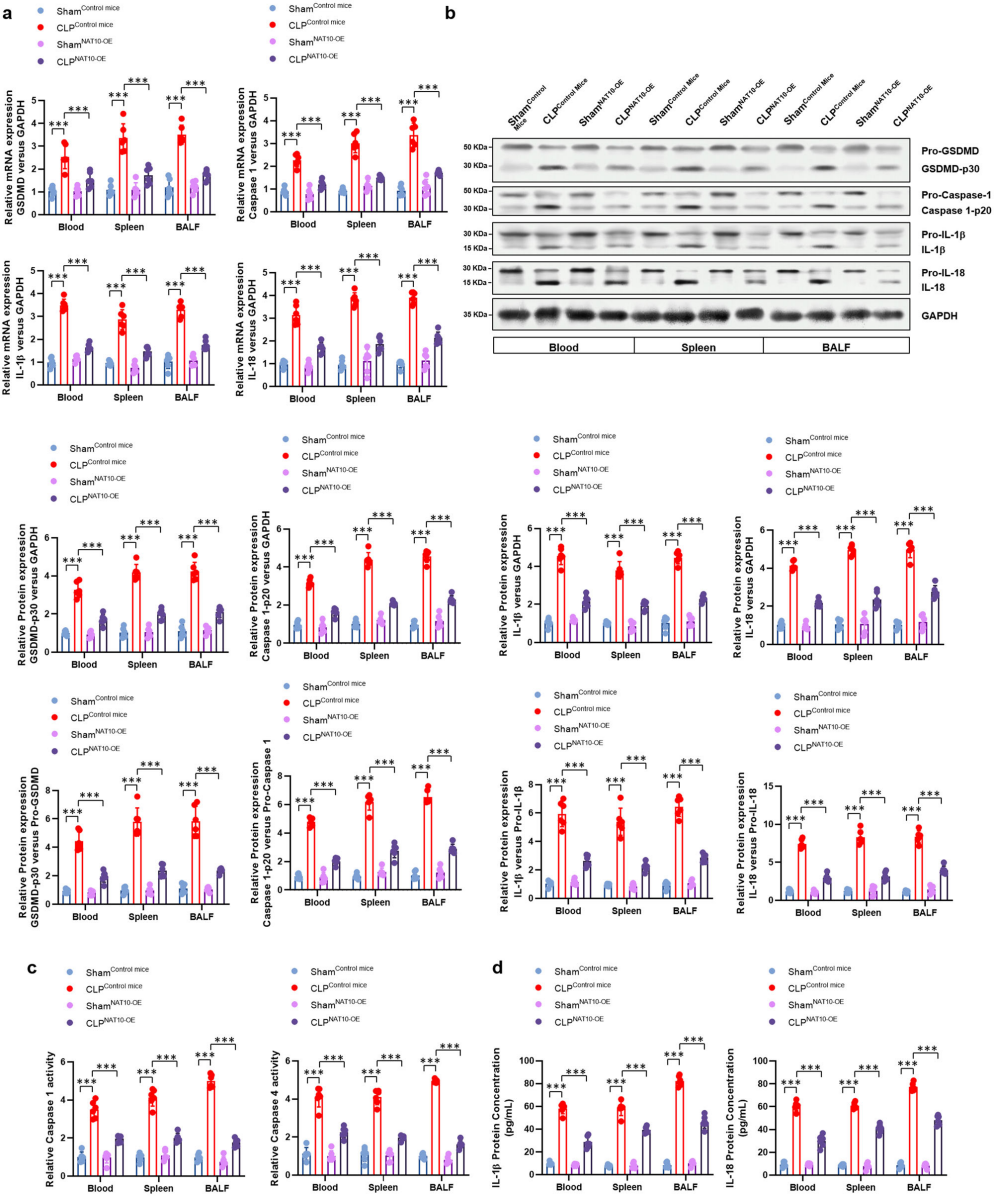
NAT10 over-expression diminished neutrophil pyroptosis in septic mice. Neutrophils were harvested from peripheral blood, spleens, and BALF from transgenic mice (MRP8Cre/Rosa26-loxP-stop-loxP-NAT10, NAT10-OE) or control mice (Rosa26-loxP-stop-loxP-NAT10, NAT10-Ctrl), either performed with CLP procedure or sham, *n* = 6 per group. **a** mRNA expression levels of *GSDMD*, *caspase-1*, *IL-1β* and *IL-18* in neutrophils were detected by real-time PCR. **b** Pyroptosis-related proteins were detected in the neutrophils isolated from sham or CLP-induced septic mice by western blot. **c** Activity of caspase 1 and caspase 4 in neutrophils of sham or CLP-induced septic mice were measured by colorimetric assay. **d** Concentration of IL-1β and IL-18 in the supernatant of neutrophil cultures were assessed by ELISA. Experiments were repeated for three times independently and representative image from one of the experiments were shown. Data are presented as dot-plots of individual experiments and mean values ±  SD. *P < 0.05, ***P < 0.001.

We then investigated the role of NAT10 insufficiency in the development of sepsis. To this end, transgenic mice with neutrophil-specific over-expression of NAT10 (MRP8Cre/Rosa26-loxP-stop-loxP-NAT10, NAT10-OE) were used in the CLP model, with Rosa26-loxP-stop-loxP-NAT10 mice as the control (NAT10-Ctrl). The over-expression of NAT10 was verified by the amplified expression level of NAT10 and RNA ac^4^C abundance in neutrophils from peripheral blood, spleens and BALF of NAT10-OE mice (Fig. 3a, b). Notably, neutrophil-specific NAT10 over-expression significantly improved the survival of septic mice (Fig. 3c) and ameliorated the lung injury (Fig. 3d, e). Next, we further investigated whether the therapeutic effects NAT10 over-expression were associated with the pyroptosis of neutrophils, which has been considered as a major driver of the development of sepsis. Neutrophils were harvested from the peripheral blood, spleens, and BALF from sham or septic mice with or without the over-expression of NAT10. It is found that pyroptosis in sepsis neutrophils was diminished in NAT10-OE mice, indicated by the downregulated expression of pyroptosis molecules and cytokines (Fig. 4a–d and Supplementary Fig. 2). Collectively, neutrophil-specific over-expression of NAT10 can ameliorate sepsis lethality by restraining neutrophil pyroptosis, indicating the potential regulatory role of NAT10 in neutrophil pyroptosis and sepsis pathogenesis.

### ULK1 is the target of NAT10-mediated mRNA acetylation (ac^4^C) in neutrophils

**Fig. 5:**
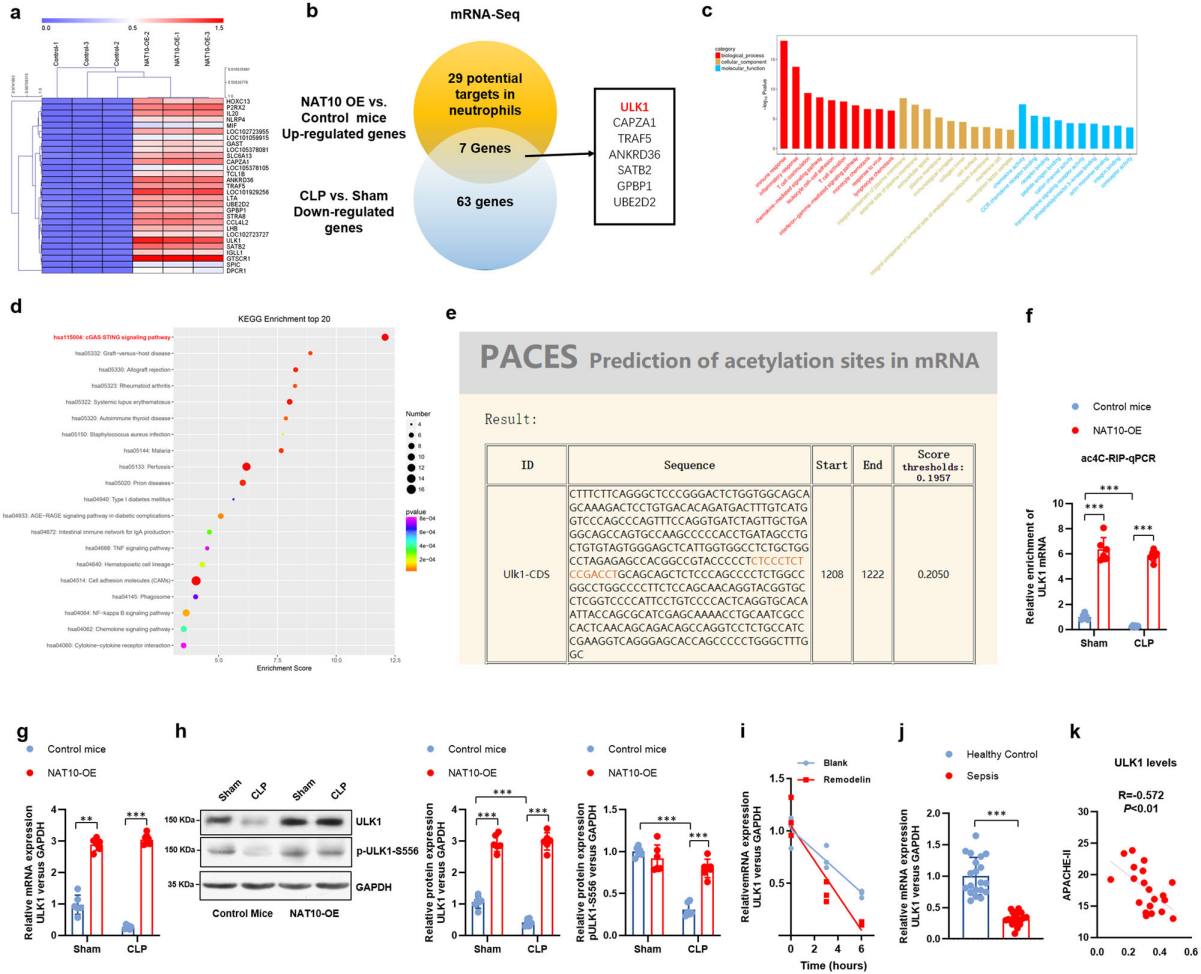
ULK1 was targeted by NAT10-mediated mRNA acetylation (ac^4^C) in neutrophils. **a** mRNA-seq was conducted in BALF neutrophils isolated from transgenic mice (MRP8Cre/Rosa26-loxP-stop-loxP-NAT10, NAT10-OE) and control mice (Rosa26-loxP-stop-loxP-NAT10, NAT10-Ctrl), wild-type sham and CLP-induced septic mice. **b** Venn diagram of the overlapped genes significantly upregulated in NAT10-OE vs NAT10-Ctrl mice and those significantly downregulated wild-type sham vs CLP-induced septic mice. **c** GO analysis indicated significant difference in biological process, cellular component, and molecular function process. **d** KEGG analysis identified significantly altered pathways in neutrophils of NAT10-OE mice, compared to neutrophils of NAT10-Ctrl mice. **e** The potential ac^4^C site *ULK1* mRNA by NAT10 was predicted via PACES. **f** Ac^4^C-RIP assay was performed in neutrophils and the alterations in ac^4^C levels of *ULK1* mRNA was detected by real-time PCR (*n* = 6). **g** BALF neutrophils were collected and the mRNA expression level of *ULK1* was determined by real-time PCR (*n* = 6). **h** BALF neutrophils were collected and the protein level of ULK1 and phosphorylated-ULK1 was determined by western blot (*n* = 6). **i** Transcription inhibition assay was conducted in BALF neutrophils to determine the enhanced degradation of *ULK1* mRNA by NAT10 inhibitor Remodelin (*n* = 3). **j**, **k** BALF neutrophils were collected from healthy control or sepsis patients (*n* = 20). **j** The expression level of and ULK1 was determined by real-time PCR. **k** The correlation between ULK1 expression and the Acute Physiology and Chronic Health Status Scoring System II (APACHE II) score was determined. Experiments were repeated for three times independently and representative image from one of the experiments were shown. Data are presented as dot-plots of individual experiments and mean values ± SD. *P < 0.05, **P < 0.01, ***P < 0.001.

To identify the targets of NAT10 in neutrophils, RNA-Seq were used to analysis the differentially expressed mRNA profiles between BALF neutrophils from NAT10-OE and control mice, and the overlapped genes that upregulated in NAT10-OE neutrophils and down-regulated in CLP neutrophils were analyzed. The result identified ULK1 as the most potential target of NAT10 in neutrophils (Fig. 5a, b), Moreover, GO and KEGG analysis identified cellular component and molecular process (Fig. 5c), and cGAS-STING signaling pathway (Fig. 5d) of ac^4^C modification upregulated genes in neutrophils of control mice, compared to that of NAT10-OE mice. The potential acetylation site of *ULK1* mRNA by NAT10 is predicted using the PACES database (Fig. 5e). To verify the NAT10-mediated ac^4^C of *ULK1* mRNA, we performed the ac^4^C-RNA immunoprecipitation (RIP) assay and detected the ac^4^C level of *ULK1* mRNA by real-time PCR. As result, neutrophils of the NAT10-OE mice showed a higher level of ac^4^C of the *ULK1* mRNA, compared with those of the control mice (Fig. 5f). NAT10-dependent ac^4^C stabilizes mRNA and boosts the translation of the target genes^18^. Accordingly, we observed the increased expression of ULK1 in BALF neutrophils of NAT10-OE mice, as well as the phosphorylated ULK1 controlling the function of ULK1 (Fig. 5g, h). Transcription inhibition assay performed in BALF neutrophils from NAT10^fl/fl^ mice further determined the enhanced degradation of *ULK1* mRNA by NAT10 inhibitor remodelin, indicating the loss of *ULK1* mRNA stability upon NAT10 inhibition (Fig. 5i). Clinically, the decreased expression of *ULK1* was also observed in BALF neutrophils of sepsis patients (Fig. 5j), which was positively correlated with the APACHE II score indicating the clinical severity (Fig. 5k).

### Over-expression of NAT10 attenuates neutrophil pyroptosis and septic lethality via the ULK1-STING-NLRP3 axis

**Fig. 6:**
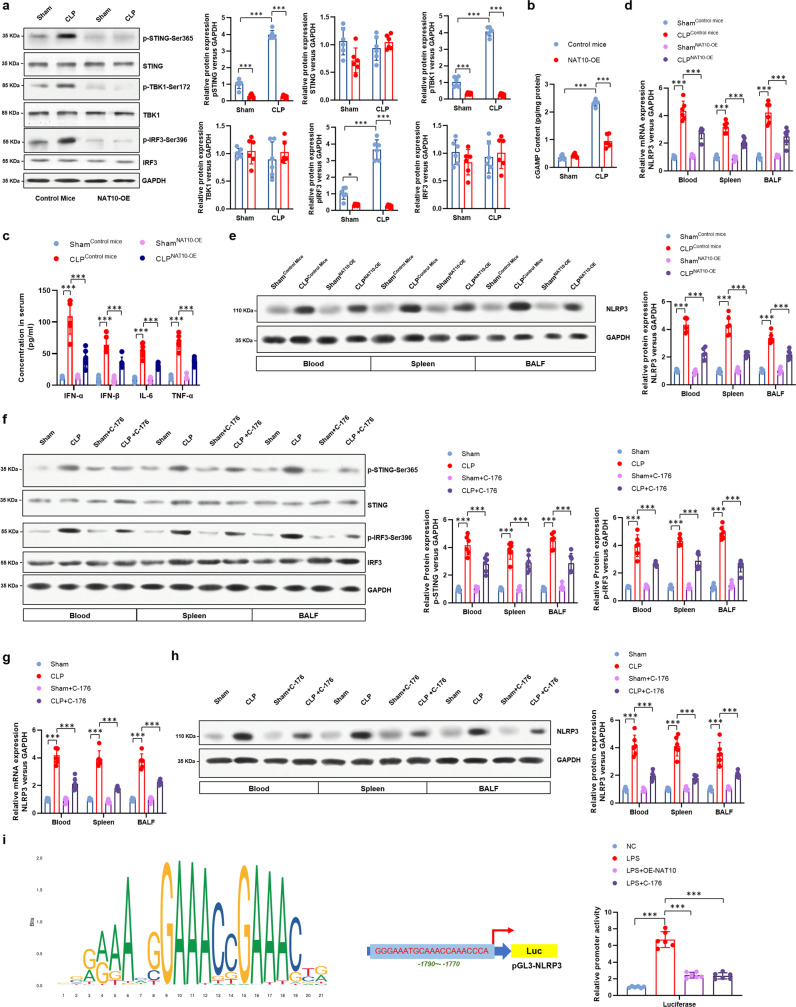
Over-expression of NAT10 in neutrophils inhibited the activation of STING and the pyroptosis-inducing NLRP3 inflammasome. Neutrophils were harvested from peripheral blood, spleens, and BALF from transgenic mice (MRP8Cre/Rosa26-loxP-stop-loxP-NAT10, NAT10-OE) or control mice (Rosa26-loxP-stop-loxP-NAT10, NAT10-Ctrl), either performed with CLP procedure or sham, *n* = 6 per group. **a** Protein level the STING-TBK1-IRF3 signaling pathway in BALF neutrophils was detected by western blot. **b** The level of cGAMP in neutrophils was evaluated by ELISA assay. **c** mRNA expression of *NLRP3* in peripheral blood, spleens, and BALF neutrophils from sham or septic mice were determined by real-time PCR. **d** The serum concentrations of IFN-α, IFN-β, IL-6, and TNF-α were detected by ELISA assay. **e** Protein level of NLRP3 in peripheral blood, spleens, and BALF neutrophils from sham or septic mice were determined by western blot. **f**–**h** STING inhibitor C-176 was *i.p*. administered to wild-type sham or CLP-induced septic mice, *n* = 6 per group. Neutrophils were harvested from peripheral blood, spleens, and BALF. **f** Activation of STING and IRF3 in neutrophils were detected by western blot. **g** mRNA expression of *NLRP3* in neutrophils was detected by real-time PCR. **h** Protein level of NLRP3 was determined by western blot. **i** Luciferase assay was conducted to examine the promoter activity of NLRP3 in HL-60-derived neutrophil-like cells with LPS-primed or unstimulated (NC). Experiments were repeated for three times independently and representative image from one of the experiments were shown. Data are presented as dot-plots of individual experiments and mean values ± SD. *P < 0.05, **P < 0.01, ***P < 0.001.

The phosphorylation of hSTING on Ser366 (mSTING on Ser365) was reported to either activate or inhibit STING function^22,24^. ULK1 has been reported to phosphorylate hSTING on Ser366 and inactivate its downstream IRF3 signaling^22^, indicating the inhibitory effect of ULK1 on STING activation. Here, we did not see any alteration of STING or cGAS expression upon NAT10 overexpression (Fig. 6a and Supplementary Fig. 3a), as well as the predicted ac^4^C site of the *cGAS* mRNA (Supplementary Fig. 3b), indicating that the regulatory effect of NAT10 on STING pathway could be mediated by ULK1. In the other hand, TBK1-mediated hSTING Ser366 phosphorylation, which is cGAMP dependent^25^, was found to activate IRF3^24,25^. In the control CLP-induced septic mice, with the low levels of NAT10 and ULK1 (Fig. 6a) while the relative high levels of cGAMP (Fig. 6b), STING-TBK1-IRF3 signaling was finally activated, as shown by the enhanced phosphorylation of mSTING at Ser365 and the p-IRF3 (Fig. 6a), as well as the production of type I IFNs and other STING-related cytokines (Fig. 6c). When NAT10 was over-expressed and ULK1 stabilized in neutrophils, the level of p-STING-Ser365 was supposed to be increased. Unexpectedly, the levels of p-STING-Ser365 was found decreased, which could be a combined effect of ULK1 and TBK1, since the diminished levels of cGAMP and activated TBK1 indicates the impairment of STING phosphorylation by TBK1 (Fig. 6a, b). STING can regulate pyroptosis in macrophages by promoting the activation of NLRP3 inflammasome, which has been playing important role in the development of sepsis^26,27^. Here, we observed the increased expression of NLRP3 in sepsis neutrophils, compared to neutrophils in sham mice. However, this is blocked by over-expressing NAT10 in neutrophils (Fig. 6d, e), consistent with the activation of STING in septic neutrophils which was restrained in NAT10 over-expressed neutrophils. Furthermore, when STING inhibitor C-176 was *i.p*. administered to wild type CLP-induced septic mice to inhibit the activation of STING-IRF3 signaling (Fig. 6f), the expression of NLRP3 in neutrophils decreased, compared to the untreated septic mice (Fig. 6g, h). The regulation of NLRP3 by STING signaling is then confirmed by the inhibited transcription activity of NLRP3 by C-176 treatment (Fig. 6i). Altogether, these data demonstrate that over-expression of NAT10 can inhibit the activation of STING and the pyroptosis-inducing NLRP3 inflammasome in neutrophils, which might be responsible for its therapeutic effect.

**Fig. 7:**
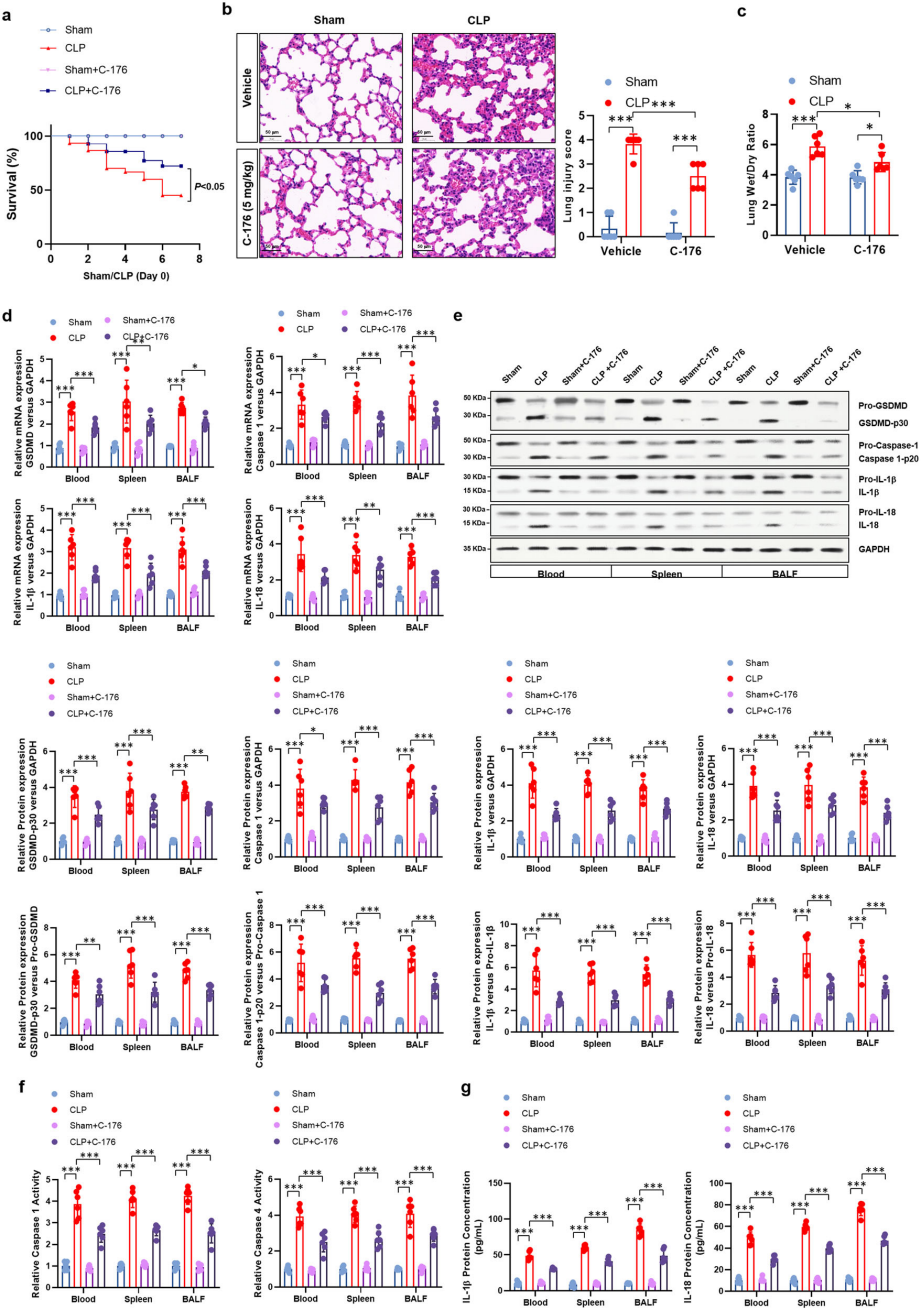
Over-expression of NAT10 attenuated septic lethality via restraining neutrophil pyroptosis regulated by the ULK1-STING-NLRP3 axis. STING inhibitor C-176 was *i.p*. administered to wild-type sham or CLP-induced septic mice, *n* = 6 per group. **a** Survival of mice were monitored. **b** Lungs were harvested and H&E staining was conducted to indicate lung injury. Lung injury score was recorded (Scale bar, 50 μm). **c** Wet/dry ratio of lung tissues were calculated. **d** mRNA expression levels of *GSDMD*, *caspase-1*, *IL-1β*, and *IL-18* in neutrophils were detected by real-time PCR. **e** Pyroptosis-related proteins were detected in the neutrophils isolated from sham or CLP-induced septic mice by western blot. **f** Activity of caspase 1 and caspase 4 in neutrophils of sham or CLP-induced septic mice were measured by colorimetric assay. **g** Concentration of IL-1β and IL-18 in the supernatant of neutrophil cultures were assessed by ELISA. Experiments were repeated for three times independently and representative image from one of the experiments were shown. Data are presented as dot-plots of individual experiments and mean values ± SD. *P < 0.05, **P < 0.01, ***P < 0.001.

**Fig. 8:**
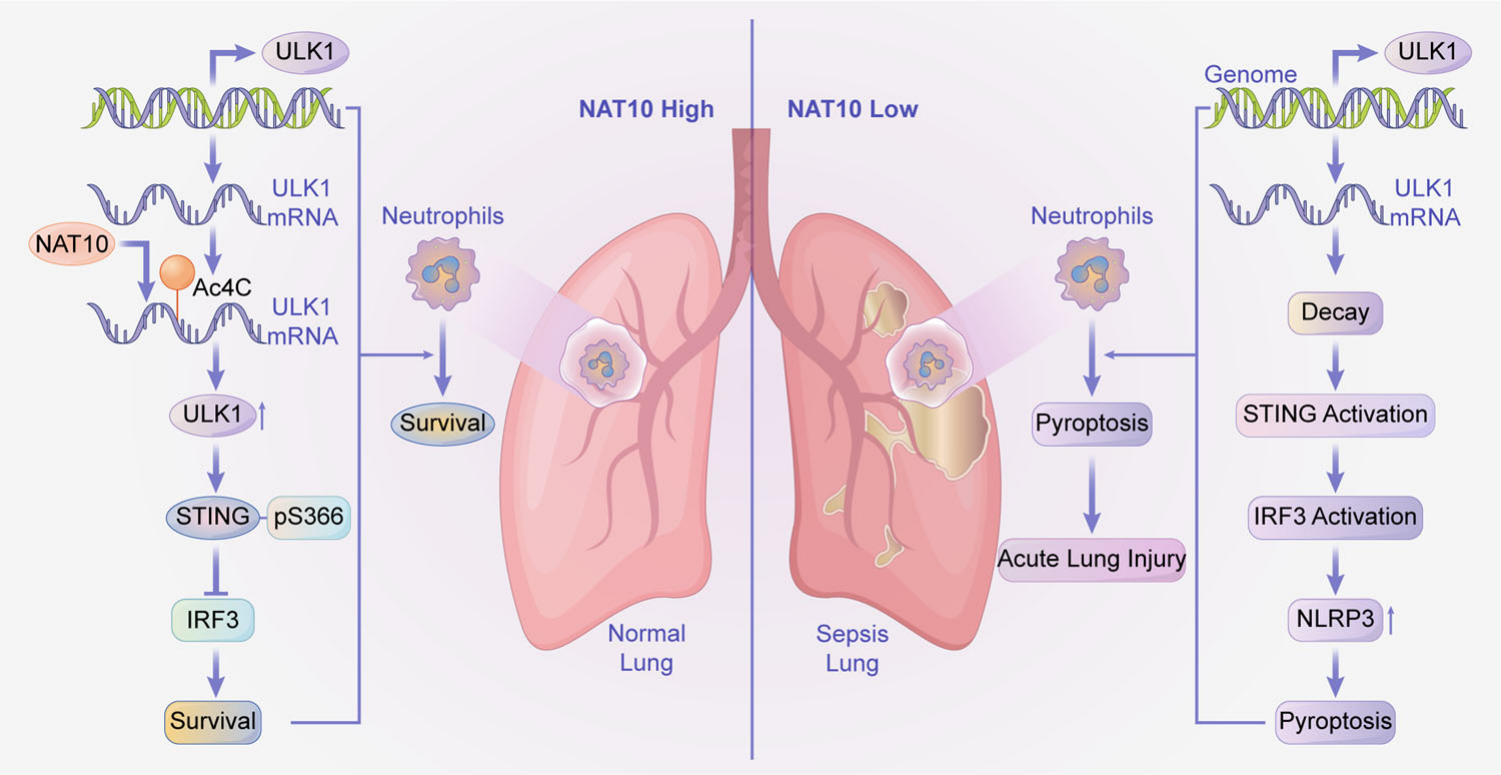
Graphical abstract. NAT10 functions as a negative regulator of neutrophil pyroptosis and a protective factor in sepsis. The downregulation of NAT10 in neutrophils contribute to the progress of sepsis by exacerbating pyroptosis in neutrophils via promoting the ULK1-STING-NLRP3 axis. The decreased expression of NAT10 resulted in the decay of ULK1 transcripts and therefore the reduced expression of ULK1. As a regulator of STING phosphorylation, the loss of ULK1 enhanced the activation of STING-IRF3 signaling and subsequently the elevated pyroptosis-inducing NLRP3 inflammasome in neutrophils, which promotes the development of sepsis. Neutrophil-specific over-expression of NAT10 can restrain pyroptosis as well as septic lethality in mice by reversing the ULK1-STING-NLRP3 axis.

Finally, to determine the role of ULK1-mediated STING-NLRP3 axis in NAT10-dependent alleviation of sepsis, STING inhibitor C-176 was administered to wild type sham or CLP-induced septic mice. The results showed inhibiting STING signaling improved the survival rate of septic mice (Fig. 7a) and ameliorated lung injury (Fig. 7b, c). More importantly, the pyroptosis in neutrophils of septic mice was remarkably restrained (Fig. 7d–g). These data suggest that STING intervention is protective to neutrophil pyroptosis and sepsis. Therefore, our data indicate that the therapeutic effect of neutrophil-specific NAT10 over-expression on sepsis by inhibiting pyroptosis is mediated by the ULK1-STING-NLRP3 axis (Fig. 8).

## Discussion

Pyroptosis is a form of programmed necrotic cell death mediated by gasdermin^28^. It has been shown that gasdermin D (GSDMD)-dependent pore formation causes the critical inflammatory event observed after pyroptosis^29^, partially by secreting pro-inflammatory cytokines such as IL-1β and IL-18. Along with the breakthrough in molecular mechanisms of pyroptosis, the pathological function of aberrant pyroptosis in diseases are getting more attention, including sepsis. Recent study has demonstrated that GSDMD-deficient animals resist LPS-induced septic shock^30^, which however, only focused on the pyroptosis in macrophages.

During sepsis, neutrophils, as the primary effector cells against infection and meanwhile critical regulator of innate and adaptive immune system, exhibits crucial roles in the pathogenesis. So far, NETosis has been considered the major form of cell deaths in neutrophils and important factor of sepsis progression^31^. Nevertheless, ample evidence indicates the unneglectable function of neutrophil pyroptotic death in sepsis^31^. At present, the mechanisms in neutrophil pyroptosis, especially the upstream molecules, remains unknown and the potential value of tarting neutrophil pyroptosis in sepsis treatment did not draw strong attention. It is assumed that neutrophil pyroptosis in sepsis controls the level of IL-1β and IL-18 and therefore the excessive inflammation. In accordance with previous studies, we observed the extensive pyroptosis in neutrophils of septic mice accompanied by massive secretion of IL-1β and IL-18. Given the important role of neutrophil pyroptosis in sepsis, we then aim to determine the upstream regulatory network of neutrophil pyroptosis in sepsis.

As the firstly recognized RNA acetyltransferase, NAT10 participates in multiple cellular processes including cell division, cellular senescence, autophagy, and DNA damage^13,32,33^. Beyond its prognostic role in multiple cancers^15,34^, a significant correlation between NAT10 expression and tumor immune infiltration was observed in a recent study^35^. However, the function of NAT10 in immune cell function remains to be clarified. Surprisingly, our current study observed a significantly downregulation of NAT10 in neutrophils from septic mice, compared to sham mice. More importantly, lower level NAT10 is also found in neutrophils from sepsis patients, compared to those from the healthy controls, which is positively correlated with disease severity. These data give us hints of the involvement of neutrophil NAT10 in sepsis. Thus, we created transgenic mice with neutrophil-specific over-expression of NAT10 to further investigate the role of NAT10. Via this murine model, we found that NAT10 functions as a negative regulator of neutrophils pyroptosis and a protective factor of sepsis, indicating its potential value in sepsis treatment via restraining neutrophil pyroptosis.

As shown by the current data and previous studies^20,36^, the cGAS-STING pathway is activated and the aberrant activation contributes to the pathogenesis. PAMPs and DAMPs derived from microbes and host tissues participate in the progression from infection to sepsis^37^. For example, a great deal of LPS is released into the blood circulation during sepsis^38^. STING is an adapter protein which can be stimulated in response to bacterial product such as CpG DNA, as well as host self-DNA including nuclear DNA and mitochondrial DNA. Therefore, it is suggested that the abundant PAMPs and DAMPs in the septic microenvironment are associated with the activation of cGAS-STING pathway in sepsis patients and CLP-induced sepsis mouse models.

NLRP3 inflammasome is the canonical inducer of pyroptosis^39^. STING has been suggested to promote NLRP3 inflammasome activation in macrophages^40^ and therefore, may contribute to the subsequent pyroptosis, which still needs to be verified. In the current study, we identified ULK1 as the target of NAT10, whose RNA stability can be augmented by NAT10-dependent ac^4^C and the expression enhanced. Interestingly, ULK1 has been found to inhibit the STING activation by phosphorylation^22^. Hence, it is reasonable to hypothesize that NAT10 can regulate pyroptosis via the ULK1-STING-NLRP3 axis, for which, data from our study can be supportive evidence.Nevertheless, it needs to be further confirmed by intervening ULK1 and NLRP3 in the context of NAT10 over-expression. NLRP3 expression is regulated by NF-κB activity, the downstream of STING. However, ULK1 phosphorylation of STING was found will not affect the NF-κB pathway^22^, therefore, there could be other mechanisms governing the ULK1 regulation on NLRP3 inflammasome instead of NF-κB. In one hand, a recent study suggests that STING can promote NLRP3 localization in ER and facilitates NLRP3 deubiquitination to activate the inflammasome^40^. On the other hand, ULK1 has been demonstrated participated in the autophagic degradation of NLRP3^41^, indicating a direct regulatory role of ULK1 on NLRP3 inflammasome besides via inhibiting STING. In addition, the possibility of non-canonical inflammasome triggered by cytoplasmic LPS in NAT10-mediated neutrophil pyroptosis should be investigated in future studies.

In conclusion, the current study described the negative regulation of NAT10 on neutrophil pyroptosis and its protective role in sepsis. In other words, the downregulation of NAT10 in neutrophils contribute to the progress of sepsis by exacerbating pyroptosis in neutrophils via promoting the ULK1-STING-NLRP3 axis. While over-expressing NAT10 in neutrophils can ameliorate lethality of sepsis. Therefore, the role and mechanism of NAT10 in neutrophil pyroptosis demonstrated in the study not only provide insights into the pathogenesis of sepsis, but also harbor the potential to prevent or treat sepsis via regulating neutrophil pyroptosis.

## Methods

### Ethnical statement

This study was approved by the Ethics Committee of Zhongshan Hospital, Fudan University (B2021-182R), and complied with the ethical standards set out in the Declaration of Helsinki. Written informed consent was obtained from patients or their relatives. All mice experiments were conducted in accordance with the guidelines the animal review committee at Zhongshan Hospital, Fudan University (Protocol license number: 2020-119).

### Patients

Twenty patients diagnosed with sepsis based on the third international consensus definition for sepsis^42^ and admitted to the ICUs were enrolled in this study. For healthy controls, twenty individuals who were admitted for evaluation of solitary pulmonary nodule without evidence of pulmonary infection were enrolled. Peripheral blood was collected within the first 48 h of diagnosis. Bronchoalveolar lavage was carried out to and bronchoalveolar lavage fluid (BALF) were collected. Demographic and clinical data, including age, gender, body mass index (BMI), comorbidities, Acute Physiology and Chronic Health Status Scoring System II (APACHE II) score and final diagnosis were recorded.

### Animals

C57BL/6 J male mice (8–10 weeks old), Rosa26-loxP-stop-loxP-NAT10 transgenic mice were obtained from Shanghai Laboratory Animal Research Center (Shanghai, China), neutrophil-specific MRP8-Cre mice were donated by Professor Ru-lin Shen. MRP8-Cre mice were crossed with Rosa26-loxP-stop-loxP-NAT10 transgenic mice (Control mice) to generate neutrophil-specific NAT10 over-expression mice (NAT10-OE mice). All animals were maintained by the Department of Laboratory Animal Science of Fudan University under a 12-h light–dark cycle and specific pathogen-free conditions.

### Sepsis model

Sepsis model was established by cecal ligation and puncture (CLP) as previously described^43^. Briefly, a 1–2-cm-long abdominal incision was made and the latter 33% of the cecum was ligated, punctured twice with a 25-gauge needle. The cecum was placed back and the abdomen was closed. Sham-operated mice underwent the same protocol without the CLP procedure. For a secondary infection, mice were then intranasally inoculated with 20 μL of *Pseudomonas aeruginosa* (PA) containing the 1 × 10^6^ CFUs of bacteria.

For STING intervention, sham or septic mice were *i.p*. administered with STING inhibitor C-176 (5 mg/kg, Med Chem Express). For pyroptosis intervention, pyroptosis inhibitor Ac-FLTD-CMK (10 mg/kg) was administered as described previously^44^. In some experiments, septic mice model was established in neutrophil-specific NAT10 over-expression (MRP8Cre/Rosa26-loxP-stop-loxP-NAT10) or control mice (Rosa26-loxP-stop-loxP-NAT10). For histopathology and cell analysis, serum, peripheral blood, spleens, and lungs were harvested.

### Neutrophil isolation and culture

Neutrophils were isolated on a Percoll (pH 8.5–9.5; Sigma-Aldrich) density gradient as described^45,46^. Neutrophils (>95% pure, >97% viable, by exclusion of trypan blue) were resuspended in RPMI 1640 medium (Flow Laboratories, Rickmansworth, UK) containing 0.15% bovine serum albumin (Sigma-Aldrich, St. Louis, MO).

### Caspase 1 and caspase 4 activity assay

The activities of Caspase 1 and Caspase 4 were detected by using colorimetric assay kits (C1101 and C1121, Beyotime, China) according to the instructions. This assay was based on the ability of caspase-1/4 to change acetyl-Tyr-Val-Ala-Asp p-nitroaniline (Ac-YVAD-pNA)/ acetyl-Leu-Glu-Val-Asp p-nitroanilide (Ac-LEVD-pNA) into the yellow formazan product p-nitroaniline (pNA). Briefly, a total of 50 μg cytosolic protein was incubated in a 96-well microtiter plate with 20 nmol Ac-YVAD-pNA/ Ac-LEVD-pNA overnight at 37 °C. The absorbance values of pNA at 405 nm (OD405) were detected by a spectrophotometer (Bio-Rad, Hercules, CA, USA).

### H&E staining

Lung tissue was removed, washed with PBS, fixed with 4% PFA, and embedded in paraffin. Formalin-fixed paraffin-embedded lung tissue sections were stained with H&E, and a histopathological examination was performed. The stained sections were analyzed under a light microscope (Carl Zeiss, Jena, Germany). The histologic injury scores were calculated according to the sum of the score for alveolar edema, alveolar hemorrhage, pulmonary interstitial thickening, and neutrophil infiltration. Each histological characteristic was evaluated on a scale from 0 to 3: 0, normal (no injury); 1, minimal (injury to 25% of the field); 2, mild (injury between 25 and 50% of the field); 3, moderate (injury between 50 and 75% of the field); and 4, severe (injury over 75% of the field).

### Lung wet-to-dry ratio

Lung inflammation were assessed by measurements of the lung wet-to-dry weight ratio. The right lung was separated, weighed (wet weight), and then dried overnight in an oven at 60 °C (dry weight). The wet weight/dry weight ratio was calculated by dividing the wet weight by the dry weight.

### Ac^4^C site prediction

The potential acetylation sites on *ULK1* mRNA was predicted by an online tool PACES (http://rnanut.net/paces/)^47^.

### Luciferase reporter assay

NLRP3 promoter activity was analyzed by luciferase assay as previously described^48^. Briefly, human NLRP3 gene promoter were inserted into the firefly luciferase vector pGL3-basic (Promega), termed pGL3-NLRP3. The human leukemia cell line (HL-60) is used as an alternative to primary neutrophils as described^49^. Briefly, HL-60 was differentiated into neutrophil-like cells in the medium of RPMI) 1640 plus l-glutamine and 25 mM HEPES (Fisher Scientific) supplemented with antibiotic/antimycotic (Invitrogen) and 15% heat-inactivated fetal bovine serum (FBS) (Invitrogen), plus Dimethyl sulphoxide (DMSO), endotoxin, and hybridoma tested (Sigma). Neutrophil-like cells were then transfected by pGL3-NLRP3 plasmid using Lipofectamine 2000 Reagent (Invitrogen). After 48 h, the cells were detected by a luciferase reporter gene assay system (Promega). Normalization of firefly luciferase activity was based on Renilla luciferase activity.

### Flow cytometry

Single-cell suspensions of the spleen and BALF of mice were prepared. Peripheral blood mononuclear cells (PBMCs) were isolated from murine blood by density gradient cell separation using Histopaque 1083 (Sigma-Aldrich) and red blood cell lysis. Cells were suspended in FACS buffer (PBS + 1% BSA + 1 mM EDTA) and incubated with mouse Fc blocker to block nonspecific binding sites and then incubated with specific antibodies for cell types identification. The stained cells were analyzed using FACSVerse and data files were analyzed using FlowJo software (Tree Star). Antibodies used in the study are APC anti-mouse CD11b Antibody (Cat #101212, Biolegend) and FITC anti-mouse Ly-6G Antibody (Cat #127605, Biolegend) with the dilution of 1:100 (Supplementary Fig. 4). The antibodies was listed in the Supplementary Table 1.

### RNA sequencing (mRNA-seq)

mRNA-seq was processed by Shanghai Biochip Co., Ltd., (Shanghai, China) according to the instructions of NEB Next Ultra RNA Library Prep Kit for Illumina (New England Bio Labs). Briefly, total RNAs were isolated from BALF neutrophils from wild type sham or septic mouse, and NAT10-OE or NAT10-Ctrl mice using Trizol reagent. Poly(A) RNA was subsequently purified and used to generate cDNA libraries. All samples were sequenced on Illumina HiSeq X Ten platform. Sequence reads were mapped to the human genome version hg38 by using Illumina sequence analysis pipeline. The average gene expression values of three independent studies were used for analysis.

### Ac^4^C RNA immunoprecipitation-qPCR

The ac^4^C RIP assay was performed using GenSeq ac4C RIP kit (GS-ET-005, Cloudseq Biotech, Shanghai, China) following manufacturer’s instructions. Briefly, the total RNA (200 µg) was randomly digested into nucleotide chains of 100–200 bp, and a mixture of 5 µg ac^4^C antibody and magnetic beads incubated with the disrupted RNA. Then, the RNAs were purified and the enrichment of *ULK1* mRNA was analyzed by RT-qPCR. Primers used in the assay was listed in the Supplementary Table 2.

### Transcription inhibition assay

BALF neutrophils were seeded in 12-well plates and treated with actinomycin D at a concentration of 20 μg/mL for 0, 2, 4, and 6 h, with or without Remodelin (20 mM). After treatment, RNA was immediately extracted, and PCR was performed. The turnover rate and half-life of the target mRNA were calculated to analyze the degradation rate.

### RNA Ac^4^C Dot Blot

The Poly(A) + RNA was denatured by heating for 5 minutes at 65 °C and then transferred to nitrocellulose membrane using a Bio-Dot device (Bio-Rad). Then the membranes were cross-linked by UV, blocked, incubated Ac^4^C antibody (Abcam), followed by incubation with second antibody (Abcam). Finally, the membrane can be displayed via a chemiluminescence system (Bio-Rad).

### Quantitative real-time PCR

Total RNA was extracted from murine tissues or cells by TRIzol reagent (Invitrogen, USA), and cDNA was extracted by an ABI High-Capacity cDNA Reverse Transcription Kit (Thermo Fisher Scientific, Waltham, MA, USA) according to the manufacturers’ instructions. Quantitative real-time PCR was performed using the ABI StepOnePlus Real-Time PCR system (Applied Biosystem, Foster City, CA, USA). GAPDH was used as an endogenous control. Data were analysed using 2^− ΔΔCT^. Primers used in the assay was listed in the Supplementary Table 2.

### Western blotting

Protein was isolated from tissues or cells, separated by 12% SDS-PAGE and transferred to PVDF membranes. Membranes were then probed with primary antibodies against target genes at 4 °C overnight, followed by incubating with HRP-conjugated anti-mouse secondary antibodies at room temperature for 1 h. Bands were developed using enhanced chemiluminescence technique (ChemiDoc XRS System, Bio-Rad Laboratories) and detected by ImageQuant LAS 4000 imager (GE Healthcare). The intensities of bands were measured using ImageJ software (Bethesda). Relative expressions of the target proteins were quantified as the ratio of the intensity of target proteins versus GAPDH in the same sample. The antibodies was listed in the Supplementary Table 1.

### Enzyme-linked immunosorbent assay

Murine whole blood was centrifuged at 3000 RPM for 15 min, and serum was collected and stored at −80 °C. Serum concentrations of IL-1β, IL-18, IFN-γ, TNF-α, IL-6, IL-8, G-CSF, and cGAMP were determined using Enzyme-linked immunosorbent assay (ELISA) kits according to manufacturer’s instructions.

### Immunofluorescence

For immunofluorescence experiment, pulmonary specimens were fixed with 4% paraformaldehyde and embedded in optimal cutting temperature compound before sectioned. Frozen sections were incubated with DAPI (Invitrogen). Immunofluorescence was assessed by immunofluorescence microscopy (Olympus) and data were collected with ImageJ software.

### Statistics and reproducibility

Data were statistically analyzed by SPSS 17.0 software. Data were presented as mean ± SEM. The mean of two independent samples was compared by Student’s *t* test, and the mean of multiple groups was compared by one-way analysis of variance. A *P* value <0.05 was considered statistically significant. In the survival study, a logarithmic scale test was used. For sample size of the animals, *n* = 20 per group for the survival analysis, *n* = 5 or 6 per group for other experiments. All experiments were repeated three times independently.

### Reporting summary

Further information on research design is available in the Nature Research Reporting Summary linked to this article.

## Supplementary information

Supplementary Information

Description of Additional Supplementary Files

Supplementary Data 1

Reporting Summary

## Data Availability

The source data for the graphs in the main figures are provided in Supplementary Data 1. The original uncropped blot/gel images of the main figures are provided in Supplementary Fig. 5. The mRNA-sequencing data was available on the National Omics Data Encyclopedia (NODE) database (https://www.biosino.org/node/project/detail/OEP003552). The other data that support the findings of this study are available from the corresponding author, upon reasonable request.
