# Author Correction: NAT10 regulates neutrophil pyroptosis in sepsis via acetylating ULK1 RNA and activating STING pathway

**DOI:** 10.1038/s42003-022-04045-w

**Published:** 2022-10-13

**Authors:** Hao Zhang, Zhaoyuan Chen, Ji’an Zhou, Jiahui Gu, Han Wu, Yi Jiang, Shenjia Gao, Yun Liao, Ruling Shen, Changhong Miao, Wankun Chen

**Affiliations:** 1 Department of Anesthesiology, Zhongshan Hospital, Fudan University; Cancer Center, Zhongshan Hospital, Fudan University, 200032 Shanghai, China; 2 Shanghai Key laboratory of Perioperative Stress and Protection, Shanghai, China; 3 Department of Respiratory and Critical Care Medicine, Huadong Hospital Affiliated to Fudan University, Shanghai, China; 4 Shanghai Medical College of Fudan University, Shanghai, China; 5 Shanghai Laboratory Animal Research Center, 201203 Shanghai, China; 6 Fudan Zhangjiang Institute, Shanghai, 201203 China

**Keywords:** Bacterial infection, Cell death and immune response

Correction to: *Communications Biology* 10.1038/s42003-022-03868-x, published online 06 September 2022.

In this article the author name Ruling Shen was incorrectly written as Rulin Shen. The original article has been corrected.

