# Supplementary material for: NAT10 regulates neutrophil pyroptosis in sepsis via acetylating ULK1 RNA and activating STING pathway
Source: Commun Biol. 2022 Sep 6;5:916. doi: 10.1038/s42003-022-03868-x (PMC9448771; doi:10.1038/s42003-022-03868-x)
Supplement: Supplementary file 1 — Supplementary Information [file 42003_2022_3868_MOESM1_ESM.pdf]

Supplementary Figure 1.

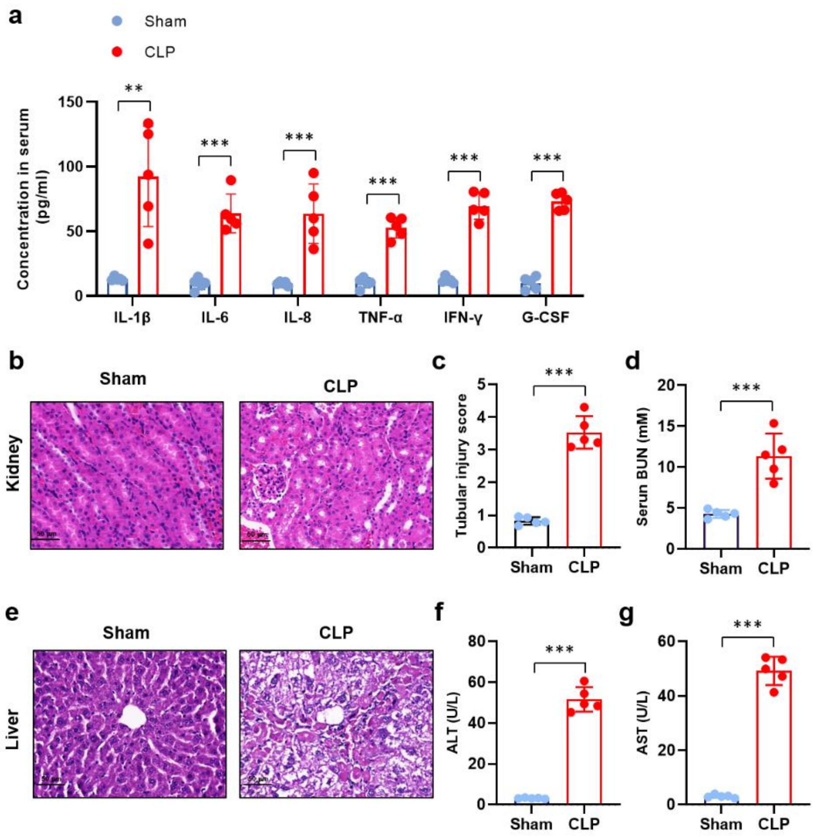

Supplementary Figure 1. Organ injuries in the CLP-induced septic mice. C57 BL/6 mice were randomly assigned to sham or CLP-induced sepsis group, n=5 per group. (**a**) The serum concentrations of IL-1b, IL-6, IL-8, TNF-a, IFN-g and G-CSF were measured by ELISA assay. (**b-c**) Kidney injury was assessed by H&E staining and the tubular injury score (Scale bar, 50 μm). (**d**) Serum concentration of BUN was detected by ELISA assay. (**e**) Liver injury was assessed by H&E staining. (**f-g**) Serum level of ALT and AST was determined by ELISA assay. Experiments were repeated for 3 times independently and representative image from one of the experiments were shown. Data are presented as dot-plots of individual experiments and mean values ± SD. *P<0.05, **P<0.01, ***P<0.001.

Supplementary Figure 2

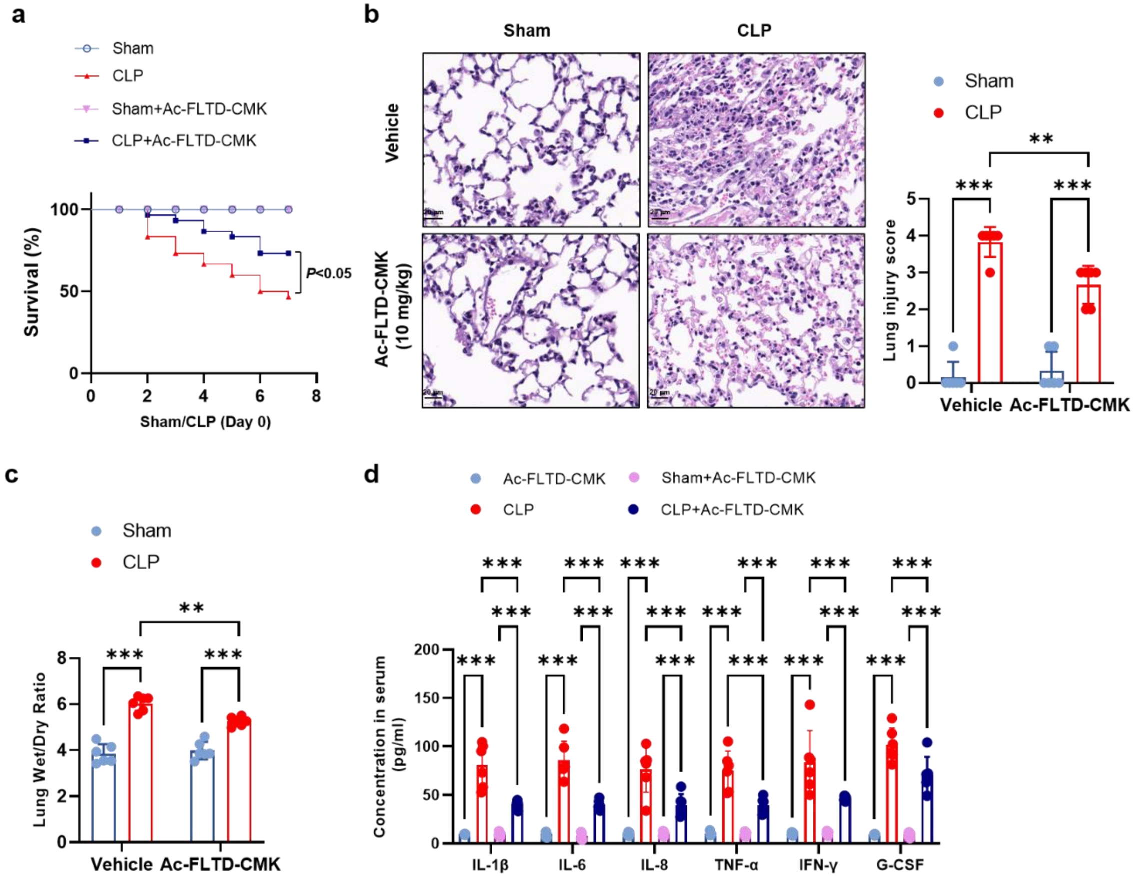

Supplementary Figure 2. The effect of pyroptosis inhibition on sepsis. C57 BL/6 mice were randomly assigned to sham or CLP-induced sepsis group and further treated with pyroptosis inhibitor Ac-FLTD-CMK (10 mg/kg) or vehicle, n=6 per group. (**a**) Survival of sham and CLP-induced septic mice without or without the treatment of Ac-FLTD-CMK. (**b**) Murine lungs were harvested, lung injury was assessed by H&E staining. Lung injury score was recorded (Scale bar, 20 μm). (**c**) Wet/dry ratio of lung tissues were calculated. (**d**) Serum concentration of cytokines were assessed by ELISA. Experiments were repeated for 3 times independently and representative image from one of the experiments were shown. Data are presented as dot-plots of individual experiments and mean values ± SD. ** $P<0.01$, *** $P<0.001$.

Supplementary Figure 3.

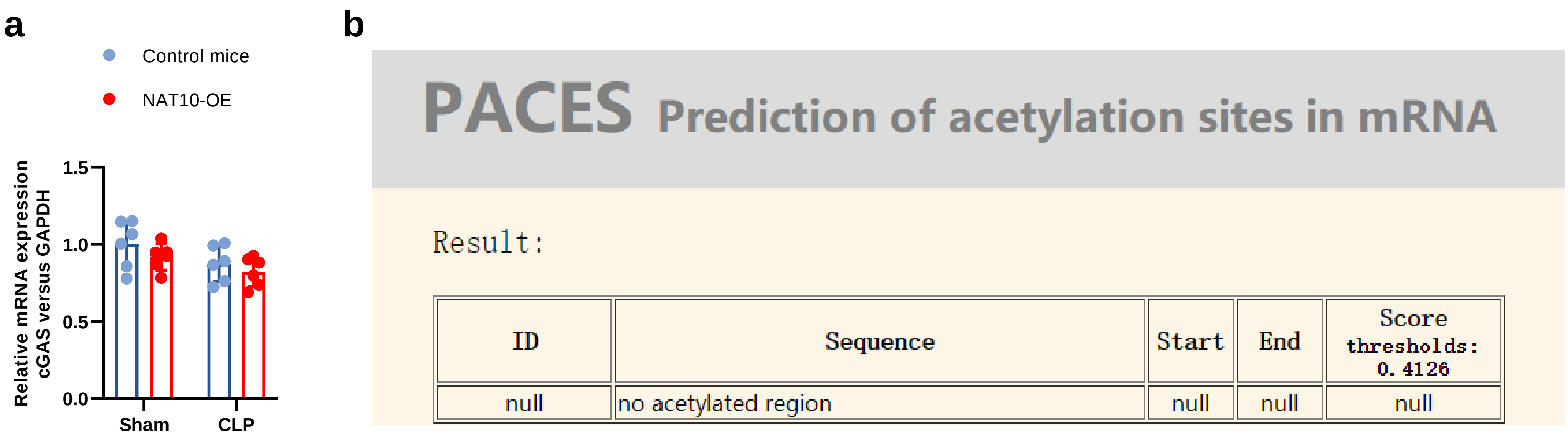

Supplementary Figure 3. Expression of *cGAS* mRNA and the predicted ac⁴C site. (**a**) BALF neutrophils were collected from NAT10-OE mice or control mice, the mRNA expression level of *cGAS* was determined by real-time PCR (n=6). Data are presented as dot-plots of individual experiments and mean values ± SD. (**b**) The potential ac⁴C site of *cGAS* mRNA by NAT10 was predicted via PACES.

Supplementary Figure 4

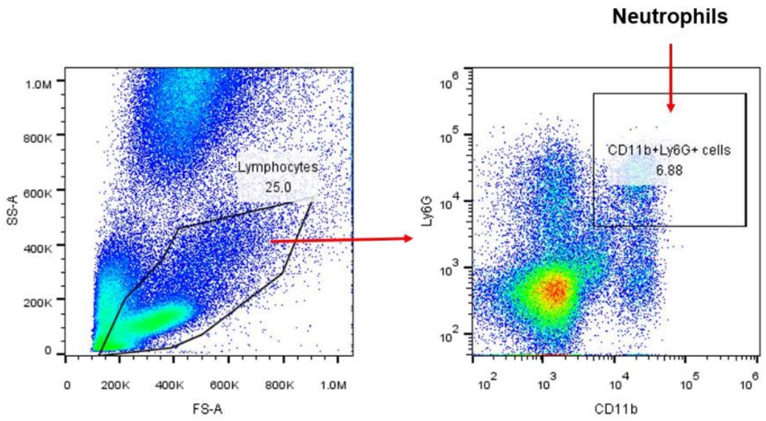

Supplementary Figure 4. Gating strategy used for flow cytometry. Lymphocytes were gated by forward. Neutrophils were gated based on CD11b and Ly6G positivity.

Supplementary Figure 5

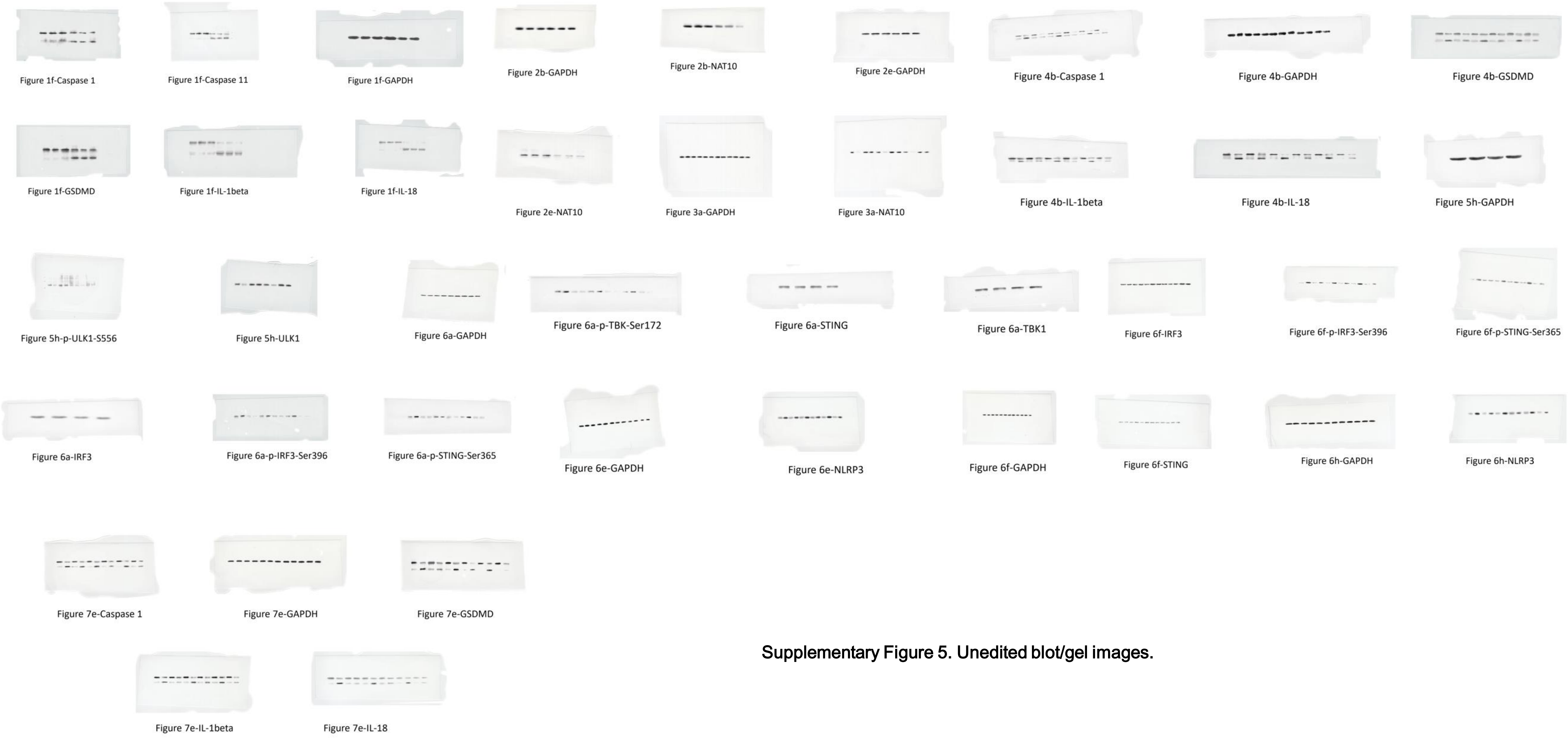

Supplementary Figure 5. Unedited blot/gel images.

Supplementary Table 1. List of antibodies.

Name	Cat. #	Manufacturer	Concentration
APC anti-mouse CD11b Antibody	101212	Biolegend	1:100
FITC anti-mouse Ly-6G Antibody	127605	Biolegend	1:150
Anti-GSDMD antibody	ab214818	Abcam	1:2500
Anti-Caspase 1 antibody	ab179515	Abcam	1:2000
Anti- Caspase 11 antibody	ab240991	Abcam	1:1000
Anti- IL-1b antibody	ab216995	Abcam	1:1000
Anti-IL-18 antibody	57058	Cell Signaling Technology	1:2000
Anti-NAT10 antibody	66548	Cell Signaling Technology	1:2000
Anti-ULK1 antibody	8054	Cell Signaling Technology	1:1500
Anti-ULK1 (phospho S556) antibody	ab203207	Abcam	1:1000
Anti-STING antibody	ab288157	Abcam	1:2000
Anti-STING-Ser365 antibody	72971	Cell Signaling Technology	1:1000
Anti-TBK1 antibody	3504	Cell Signaling Technology	1:2000
Anti-TBK1-Ser172 antibody	5483	Cell Signaling Technology	1:2000
Anti-IRF3 antibody	4302	Cell Signaling Technology	1:1000
Anti-IRF3-Ser396 antibody	83611	Cell Signaling Technology	1:1500
Anti-NLRP3 antibody	15101	Cell Signaling Technology	1:2000
HRP-linked Anti-mouse IgG	7076	Cell Signaling Technology	1:5000
HRP-linked Anti-Rabbit IgG	7074	Cell Signaling Technology	1:5000
HRP-linked Anti-GAPDH antibody	8840	Cell Signaling Technology	1:6000

Supplementary Table 2. List of primers used in quantitative real-time PCR.

Name	Forward (5'-3')	Reverse (5'-3')
NAT10	GCCCGGATAACCTCCACACG	CCTGGCCCAGTTTCACAGCA
GSDMD	GAGGTAAGCGGCAGCAGAGG	CACTGCAGCCCCACCAGAAA
Caspase 1	TGGACAAGGCACGGGACCTA	AGGGTCCCAGTCAGTCCTGG
Caspase 11	GATGTCCAGCCGTGGTCGAG	CCCTGATCTGTCTGCCGCTG
IL-1b	TGCTGGTGTGTGACGTTCCC	GGTGGGTGTGCCGTCTTTCA
IL-18	CCGACTTCACTGTACAACCGCA	GGTCTGGGGTTCACTGGCAC
ULK1	GCTTGCGACTTCAGCGCTTC	CCCTCCAGCAATAGCAGGGC
ULK1 (for ac4C-RIP-qPCR)	CCCAGCCCAGTTTCCAGGTG	TGTCTGCTGTTGAGTGGGCG
NLRP3	GGAGCGGGAGCATGAACTCC	ACCACTGTGTGCACAGGCTC
cGAS	AGGCGCGGAAAGTCGTAAGG	GCCAGTGTTCAGCTGCTCCA
GAPDH	TGTGGATGGCCCCTCTGGAA	TGACCTTGCCCACAGCCTTG
