# Supplementary material for: NAT10 regulates neutrophil pyroptosis in sepsis via acetylating ULK1 RNA and activating STING pathway
Source: Commun Biol. 2022 Sep 6;5:916. doi: 10.1038/s42003-022-03868-x (PMC9448771; doi:10.1038/s42003-022-03868-x)
Supplement: Supplementary file 2 — Description of Additional Supplementary Files [file 42003_2022_3868_MOESM2_ESM.pdf]

# Description of Additional Supplementary Files

**File name:** Supplementary Data 1
**Description:** The source data for the graphs in the main figures.
